# Clinical characteristics of pregnant women with COVID-19 and infection outcomes in one of the largest cities in the Brazilian Amazon

**DOI:** 10.1186/s12879-024-09982-x

**Published:** 2024-10-18

**Authors:** Ana Paula Figueiredo de Montalvão França, Jenephy Thalita Rosa Paixão, Ricardo Roberto de Souza Fonseca, Rogério Valois Laurentino, Luana Gabriella Figueiredo de Montalvão Leite, Amanda Souza França Veras, Francisco Jordano da Silva Feitosa Ribeiro, Pablo Fabiano Moura das Neves, Luís Fábio Magno Falcão, Ana Carla Figueiredo de Montalvão Serrão, Aldemir Branco Oliveira-Filho, Luiz Fernando Almeida Machado

**Affiliations:** 1 Biology of Infectious and Parasitic Agents Post-Graduate Program, Federal University of Pará, Belém, Pará Brazil; 2 Virology Laboratory, Institute of Biological Sciences, Federal University of Pará, Belem, Pará Brazil; 3 School of Medicine, University Center of State of Pará, Belém, Pará Brazil; 4 School of Physiotherapy, State University of Pará, Belém, Pará Brazil; 5 School of Nursing, Amazon University, Belém, Pará Brazil; 6 Study and Research Group on Vulnerable Populations, Federal University of Pará, Bragança, Pará Brazil

**Keywords:** COVID-19, Pandemic, Maternal death, Women’s health, Brazil

## Abstract

**Background:**

Pregnancy can be a risk factor for the development of more severe COVID-19 with a possible increase in the risk of complications during pregnancy/birth and adverse neonatal outcomes. This study aimed to describe and analyze the clinical and epidemiological aspects of SARS-CoV-2 infection in women in the perinatal period attended in the city of Belém, northern region of Brazil.

**Methods:**

This is a clinical, observational, analytical, and cross-sectional study with a quantitative approach, conducted at the Santa Casa de Misericórdia do Pará Foundation (FSCMPA). It included 230 pregnant women hospitalized at FSCMPA with a positive SARS-CoV-2 RT-PCR molecular test between April 2020 and June 2022. Clinical and epidemiological information (origin, gestational age, prenatal care, comorbidities, birth complications, and chest tomography) were obtained from medical records, and correlation was made between the types of cases (mild, moderate, and severe) and maternal outcome. The chi-square test and G test were used to assess the possibility of association between variables.

**Results:**

Evidence of association was observed between the severity of COVID-19 and the following parameters: gestational age, specific pregnancy comorbidities, baby and maternal death, birth complications, and prematurity. Dyspnea, headache, anosmia, odynophagia, diarrhea, and chest pain were the symptoms most related to disease aggravation. The maternal mortality rate in the study was 8.7%.

**Conclusion:**

Specific pregnancy-related and pre-existing comorbidities associated with SARS-CoV-2 infection directly contribute to the worsening clinical condition, leading to complications such as prematurity, fetal, and maternal death.

## Introduction

Numerous studies have identified that Coronavirus Disease (COVID-19) most critical disease comorbidities and risk factors were advanced age, male gender, obesity, smoking, hypertension, diabetes, hematologic, renal, cardiovascular, respiratory diseases, and pregnancy [1–4]. Evidence supports that pregnancy is a risk factor for severe illness associated with Severe Acute Respiratory Syndrome Coronavirus 2 (SARS-CoV-2), revealing that pregnant women are more likely to be admitted to the Intensive Care Unit (ICU), require mechanical ventilation, and die than non-pregnant women, as well as an increased risk of complications during pregnancy/birth and adverse neonatal outcomes [5, 6].

The clinical manifestations of pregnant women with COVID-19 can vary from asymptomatic cases, mild symptoms such as fever, general discomfort, cough, sore throat, chest pain, chills, myalgia, and diarrhea to the most severe cases, which include heart failure, requiring mechanical ventilation, organ failure, and sepsis [7, 8], especially in women who are infected with the virus in the third trimester of pregnancy.

In theory, COVID-19 is worsened in pregnant women than non-pregnant women due to significant anatomical and physiological changes that happens during pregnancy to nurture and development of fetus, also COVID-19’s severity may be increased if pregnancy was associated with comorbidities, such as diabetes, cardiopathies or chronic lung disease. Between the main changes to occur during pregnancy are in cardiovascular and respiratory systems, in the cardiovascular system there is a peripheral vasodilatation mediated by upregulated nitric oxide synthesis, estradiol and vasodilatory prostaglandins, which results in a 25–30% decreased resistance of the vascular system and to compensate this peripheral vasodilatation, the cardiac output naturally increases by around 40% during pregnancy, also increasing the stroke volume due to the early increase in ventricular wall muscle mass and end-diastolic volume and increases even more during labor and fetus delivery [9–11].

Factors such as social determinants exacerbate the pandemic scenario in the Amazon region, where SARS-CoV-2 infection takes on unequal faces, depicted by the socio-economic, geographical, and environmental conditions of the region. At the onset of the pandemic, the Northern Region experienced the most severe epidemiological situation in Brazil, with the states of Amazonas and Pará presenting the highest numbers of confirmed cases, deaths, lethality, and hospitalizations due to severe acute respiratory syndrome (SARS) caused by COVID-19 [12]. There are limited reports on the clinical impacts and outcomes of COVID-19 during pregnancy with and without pre-existing clinical conditions and comorbidities in emerging regions, such as the Brazilian Amazon, which historically face public health challenges with higher fertility rates, especially among younger women. Therefore, information regarding maternal mortality during the COVID-19 pandemic will help to decrease these fatality rates [13, 14]. This study aims to describe the clinical and epidemiological aspects of SARS-CoV-2 infection in women during the perinatal period in a public maternity hospital in the Brazilian Amazon.

## Materials and methods

### Study design and area characterization

This is an exploratory, descriptive and cross-sectional study with a quantitative approach, which evaluated pregnant women hospitalized at Santa Casa de Misericórdia do Pará Foundation (FSCMPA), located in the city of Belém, in Pará state, in Amazon region of Brazil. During the COVID-19 pandemic in Pará state, there were around 898.496 confirmed COVID-19 cases and 19.244 deaths due to COVID-19 [15], of which 397 were deaths of pregnant women due to COVID-19 between 2020 and 2022 according Brazilian Obstetric Observatory [16], this high mortality rate among pregnant women are probably due to geographic conditions of Pará state, which causes difficulties for the population, especially pregnant women, to access structured public health services due to lower levels of industrialization, higher rates of poverty and concentration of specialized services in urban areas, such as FSCMPA.

The FSCMPA is the oldest healthcare institution (373 years) and one of the largest maternal and child hospital units (496 beds) in the Brazilian Amazon. It provides medium and high complexity care services, acting on spontaneous demand or referrals from the 144 municipalities of the state of Pará. The FSCMPA has been a reference in women’s and children’s health care in the Brazilian Amazon and was designated as a backup hospital for severe cases of COVID-19 during the critical phase of the pandemic. This study was conducted following the guidelines of the Helsinki Declaration and approved by the Research Ethics Committee of FSCMPA, under approval number 2.174.033.

## Sample size and sampling procedure

The sample size determination was based on the estimated prevalence of COVID-19 of pregnant women in Brazil, between 2020 and 2022, which had 22.000 pregnant women COVID-19 infected as population (10.4%) and it was established the sample error (ε) as 5%, and test power was assumed of 95% resulting in a minimum sample size of 225 participants.

In total, 230 pregnant women with COVID-19 hospitalized at FSCMPA participated in the study. The classification of COVID-19 in pregnant women followed the case definition proposed by the Ministry of Health [17]: (i) mild case: cough, sore throat, or runny nose, followed or not by anosmia, ageusia, diarrhea, abdominal pain, fever, chills, myalgia, fatigue, and/or headache; (ii) moderate case: persistent cough and daily persistent fever, adynamia, prostration, hyporexia, diarrhea, and pneumonia without signs or symptoms of severity; (iii) severe case: Severe Acute Respiratory Syndrome (SARS) presenting with dyspnea/respiratory discomfort or persistent chest pressure or oxygen saturation less than 95% in ambient air or bluish discoloration of lips or face.

Based on the COVID-19 classification of Ministry of Health, pregnant women were divided into 3 groups according to the severity of the infection, G1: mild cases; G2: moderate cases and G3: severe cases. The inclusion criteria were all pregnant women of any gestational age attending to FSCMPA; to reside in the Pará state at the time of the study and sign the written consent term. The exclusion criteria were patients with severe morbidities who were not in a condition to participate, pre-existing psychiatric disorders, patients not willing to participate in the study or sign the written consent term.

## Obtaining clinical-epidemiological information

Data collection was performed through a search in the electronic medical record system of FSCMPA, where a report with all the data of patients admitted to clinical or intensive care units with symptoms of influenza-like illness from April 2020 to June 2022 was obtained. Pregnant women with a positive result for SARS-CoV-2 using real-time reverse transcriptase-polymerase chain reaction (RT-PCR) were identified in the report. Subsequently, data from these women, such as medical records and examination reports, were accessed and used to present the clinical-epidemiological information in this study. All pregnant women were followed from admission, during the hospitalization period, until discharge (clinical improvement, transfer to another hospital, or death).

### Data analysis

All data collected in this study were entered into a spreadsheet in Excel and subsequently converted into a BioEstat file. The clinical and epidemiological variables of pregnant women, types of COVID-19 cases and maternal outcomes were presented using descriptive statistics, using simple frequencies and percentages; in the present study, the variables Age, municipality, ethnicity, gestational age, prenatal care, comorbidities were considered as independent, and the variables maternal clinical outcome, labor complications, lung impairment rate (%) as dependent. The variables were grouped into classes (frequency distribution) or categories (contingency tables). The chi-square test and G test were used to assess the possibility of association between variables. Statistical analysis of the data was conducted using Bioestat 5.3 software, adopting a significance level of 5%.

## Results

In total, 295 (100%) pregnant women with positive results for COVID-19 by RT-PCR were initially treated at FSCMPA. However, 40 (61.53%) patients were excluded from this study because they were not admitted to the FSCMPA, 20 (30.76%) due to the absence of data in the electronic medical record system of this public health institution and 5 (7.71%) due to transfer to another hospital during the COVID-19 pandemic, leaving a total of 65/295 (22.03%) excluded. In the final sample, *n* = 230 (77.97%) were obtained, the average age was 28 years. Most pregnant women came from municipalities Pará state countryside, as Marabá, Parauapebas, Altamira and Santarém and had self-declared mixed ethnicity, we could observe the predominance of COVID-19 severe cases among pregnant women with self-declared mixed ethnicity, from countryside and age between 31 and 39 years old (Table 1).

**Table 1:** Epidemiological profile of pregnant and postpartum women with a positive test for SARS Cov-2 and case types from April/2020 to June/2022

Age range (years)	Total (N = 230)	%	CI 95%	Light (N = 36)	%	CI 95%	Mild (N = 16)	%	CI 95%	Heavy (N = 178)	%	CI 95%	*P-valor*
< 19	23	10,0	0.061 (6.1%) − 0.139 (13.9%)	4	11,11	0.008 (0.8%) − 0.214 (21.4%)	0	0,00	0.00%	19	10,67	0.061 (6.1%) − 0.152 (15.2%)	0,2257*
Between 20 and 29	111	48,26	0.418 (41.8%) − 0.418 (41.8%)	19	52,78	0.365 (36.5%) − 0.691 (69.1%)	7	43,75	0.2835 (28.35%) − 0.5915 (59.15%)	85	47,75	0.404 (40.4%) − 0.551 (55.1%)	
Between 31 and 39	85	36,96	0.307 (30.7%) − 0.432 (43.2%)	10	27,78	0.131 (13.1%) − 0.424 (42.4%)	9	56,25	0.4090 (40.9%) − 0.7160 (71.6%)	66	37,08	0.300 (30.0%) − 0.442 (44.2%)	
³ 40	11	4,78	0.020 (2.0%) − 0.075 (7.5%)	3	8,33	0.007 (0.7%) − 0.174 (17.4%)	0	0,00	0.00%	8	4,49	0.015 (1.5%) − 0.075 (7.5%)	
**Origin**													
State’s Capital	92	40,0	0.337 (33.7%) − 0.463 (46.3%)	12	33,33	0.179 (17.9%) − 0.487 (48.7%)	7	43,75	0.2835 (28.35%) − 0.5915 (59.15%)	73	41,01	0.338 (33.8%) − 0.482 (48.2%)	0,6583*
Interior	138	60,0	0.537 (53.7%) − 0.663 (66.3%)	24	66,67	0.513 (51.3%) − 0.821 (82.1%)	9	56,25	0.4090 (40.9%) − 0.7160 (71.6%)	105	58,99	0.518 (51.8%) − 0.662 (66.2%)	
**Ethnicity**													
Brown	218	94,78	0.919 (91.9%) − 0.977 (97.7%)	35	97,22	0.9621(95.21%) − 0.9823 (92.23%)	16	100,0	100%	167	93,82	0.903 (90.3%) − 0.974 (97.4%)	0,4025*
White	6	2,61	0.005 (0.5%) − 0.047 (4.7%)	0	0,0	0.00%	0	0,0	0.00%	6	3,37	0.007 (0.7%) − 0.060 (6.0%)	
Black	6	2,61	0.005 (0.5%) − 0.047 (4.7%)	1	2,78	0.0177 (1.77%) − 0.0379 (3.79%)	0	0,00	0.00%	5	2,81	0.0166(1.66%) − 0.0432 (4.32%)	

A higher number of severe cases of COVID-19 was evidenced in the late term of pregnancy (between 41 and 42 weeks), with significant differences observed at the 5% significance level for all studied variables except for the prenatal care attendance. A statistical association between the variables gestational age and severity of COVID-19 was observed (p-value < 0.05). Another significant association was found between the disease and specific pregnancy comorbidities (preeclampsia, eclampsia and hemolysis, elevated liver enzymes and low platelets-HELLP syndrome) and the overlap of other pre-existing conditions (chronic arterial hypertension and diabetes; p-value < 0.05), the most severe cases were associated to fetal and maternal death (p-value < 0.05). Birth complications also showed significant association to maternal and fetal deaths (p-value < 0.05), additionally, when considering birth complications associated with fetal prematurity and intrauterine fetal death, as well as with chest tomography, an association between the percentage of pulmonary involvement and severity in the patients’ evolution was found (p value < 0.05). Thus, greater extents of lung involvement were evidenced in severe cases (Table 2).

**Table 2:** Clinical profile of pregnant and postpartum women with a positive test for SARS Cov-2 and case types from April/2020 to June/2022

Gestational Age	Light (N = 36)	%	CI 95%	Mild (N = 16)	%	CI 95%	Heavy (N = 178)	%	CI 95%	P-valor
1st Quarter	1	2,78	0.0182 (1.82%) − 0.0374 (3.74%)	0	0,00%	0.00%	8	4,49%	0.015 (1.5%) − 0.075 (7.5%)	0,0386* (b)
2nd Quarter	3	8,33	0.0273 (2.73%) − 0.1393 (13.93%)	4	25,00%	0.0805 (8.05%) − 0.4195 (41.95%)	47	26,40%	0.199 (19.9%) − 0.329 (32.9%)	
3rd Quarter	32	88,89	0.7899 (78.99%) − 0.9879 (98.79%)	12	75,00%	0.5805 (58.05%) − 0.9195 (91.95%)	123	69,10%	0.623 (62.3%) − 0.759 (75.9%)	
**Prenatal**	**Light (N = 36)**	**%**		**Mild (N = 16)**	**%**		**Heavy (N = 178)**	**%**		*P-valor*
Yes	30	83,33	0.7352 (73.52%) − 0.9314 (93.14%)	15	93,75%	0.8428 (84.28%) − 1 (100%)	155	87,08%	0.822 (82.2%) − 0.920 (92.0%)	***0,5556***
No	6	16,67	0.0686 (6.86%) − 0.2648 (26.48%)	1	6,25%	0 (0.0%) − 0.1572 (15.72%)	23	12,92%	0.080 (8.0%) − 0.178 (17.8%)	
**Comorbidities**	**Light (N = 36)**	**%**		**Mild (N = 16)**	**%**		**Heavy (N = 178)**	**%**		*P-valor*
None	18	50,00	0.337 (33.7%) − 0.663 (66.3%)	13	81,25	0.5969 (59.69%) − 1.0 (100%)	86	48,31	0.410 (41.0%) − 0.557 (55.7%)	0,0003* (b)
Pregnancy-specific	16	44,44	0.2893 (28.93%) − 0.5995 (59.95%)	1	6,25	0 (0.0%) − 0.1572 (15.72%)	41	23,03	0.168 (16.8%) − 0.292 (29.2%)	
Of pregnancy superimposed on other pre-existing	2	5,56	0 (0%) − 0.1446 (14.46%)	1	6,25	0 (0.0%) − 0.1572 (15.72%)	15	8,43	0.043 (4.3%) − 0.125 (12.5%)	
Pre-existing	0	0,00	0.00%	1	6,25	0 (0.0%) − 0.1572 (15.72%)	36	20,22	0.143 (14.3%) − 0.261 (26.1%)	
**Mother’s outcome**	**Light (N = 36)**	**%**		**Mild (N = 16)**	**%**		**Heavy (N = 178)**	**%**		*P-valor*
Discharge	36	100,00	100%	16	100,00	1 (100%)	158	88,76	0.841 (84.1%) − 0.934 (93.4%)	0,0045* (b)
Death	0	0,00	0.00%	0	0,00	0 (0.0%)	20	11,24	0.066 (6.6%) − 0.159 (15.9%)	
**Birth complications (1)**	**Light (N = 30)**	**%**		**Mild (N = 10)**	**%**		**Heavy (N = 122)**	**%**		*P-valor*
None	18	60,00	0.337 (33.7%) − 0.663 (66.3%)	4	40,00	0.0963 (9.63%) − 0.7037 (70.37%)	63	51,64	0.428 (42.8%) − 0.605 (60.5%)	0,0466* (b)
Intrauterine Fetal Death	3	10,00	0.0273 (2.73%) − 0.1393 (13.93%)	2	20,00	0.0482 (4.82%) − 0.4482 (44.82%)	2	1,64	0.011 (1.1%) − 0.088 (8.8%)	
Prematurity	9	30,00	0.1158 (11.58%) − 0.3842 (38.42%)	4	40,00	0.0963 (9.63%) − 0.7037 (70.37%)	57	46,72	0.379 (37.9%) − 0.556 (55.6%)	
**Range (1st Chest CT - %) (2)**	**Light (N = 19)**	**%**		**Mild (N = 14)**	**%**		**Heavy (N = 155)**	**%**		*P-valor*
< 20%	10	52,63	0.3727 (37.27%) − 0.6799 (67.99%)	3	21,43	0.0020 (0.2%) − 0.4706 (47.06%)	20	12,90	0.076 (7.6%) − 0.182 (18.2%)	0,0013* (b)
Between 20% and 40%	6	31,58	0.1851 (18.51%) − 0.4465 (44.65%)	4	28,57	0.0169 (1.69%) − 0.5545 (55.45%)	49	31,61	0.243 (24.3%) − 0.389 (38.9%)	
>=40%	3	15,79	0.0058 (0.58%) − 0.3100 (31%)	7	50,00	0.238 (23.8%) − 0.762 (76.2%)	86	55,48	0.477 (47.7%) − 0.633 (63.3%)	

Overall, we can observe an association between several clinical manifestations of pregnant and postpartum women and the severity of disease, considering the classification adopted for COVID-19 cases (Table 3).

**Table 3:** Clinical manifestations of pregnant and postpartum women with a positive test for SARS Cov-2 and case types from April/2020 to June/2022

Symptoms	Light (N = 36)	%	CI 95%	Mild (N = 16)	%	CI 95%	Heavy (N = 178)	%	CI 95%	*P-valor*
Cough – Yes	26	72,22	0.514 (51.4%) − 0.930 (93%)	14	87,50	0.769 (76.9%) − 0.981 (98.1%)	157	88,20	0.835 (83.5%) − 0.929 (92.9%)	***0,0678 (b)***
Cough – No	10	27,78	0.131 (13.1%) − 0.424 (42.4%)	2	12,50	0.0787 (7.87%) − 0.1712 (17.12%)	21	11,80	0.071 (7.1%) − 0.165 (16.5%)	
Fever – Yes	19	52,78	0.298 (29.8%) − 0.760 (76%)	12	75,00	0.5805 (58.05%) − 0.9195 (91.95%)	112	62,92	0.558 (55.8%) − 0.700 (70.0%)	***0,2797 (b)***
Fever – No	17	47,22	0.283 (28.3%) − 0.739 (73.9%)	4	25,00	0.0805 (8.05%) − 0.4195 (41.95%)	66	37,08	0.300 (30.0%) − 0.442 (44.2%)	
Dyspnea – Yes	0	0,00	0 (0.0%)	0	0,00	0 (0.0%)	167	93,82	0.903 (90.3%) − 0.974 (97.4%)	0,0001* (b)
Dyspnea – No	36	100,00	1 (100%)	16	100,00	1 (100%)	11	6,18	0.026 (2.6%) − 0.097 (9.7%)	
Myalgia – Yes	9	25,00	0.109 (10.9%) − 0.391 (39.1%)	5	31,25	0.1436 (14.36%) − 0.4814 (48.14%)	38	21,35	0.153 (15.3%) − 0.274 (27.4%)	***0,6342 (b)***
Myalgia – No	27	75,00	0.609 (60.9) − 0.891 (89.1%)	11	68,75	0.4950 (49.5%) − 0.8799 (87.9%)	140	78,65	0.726 (72.6%) − 0.847 (84.7%)	
Headache – Yes	4	11,11	0.011 (1.1%) − 0.211 (211%)	7	43,75	0.2835 (28.35%) − 0.5915 (59.15%)	39	21,91	0.158 (15.8%) − 0.280 (28.0%)	0,0363* (b)
Headache – No	32	88,89	0.812 (81.2%) − 0.966 (96.6%)	9	56,25	0.4090 (40.9%) − 0.7160 (71.6%)	139	78,09	0.720 (72.0%) − 0.842 (84.2%)	
Anosmia – Yes	4	11,11	0.011 (1.1%) − 0.211 (211%)	6	37,50	0.1364 (13.64%) − 0.6136 (61.36%)	53	29,78	0.231 (23.1%) − 0.365 (36.5%)	0,0302*(b)
Anosmia – No	32	88,89	0.812 (81.2%) − 0.966 (96.6%)	10	62,50	0.3864 (38.64%) − 0.8636 (86.36%)	125	70,22	0.635 (63.5%) − 0.769 (76.9%)	
Ageusia – Yes	0	0,00	0 (0.0%)	2	12,50	0.0787 (7.87%) − 0.1712 (17.12%)	10	5,62	0.022 (2.2%) − 0.090 (9.0%)	***0,0754 (b)***
Ageusia – No	36	100,00	1 (100%)	14	87,50	0.769 (76.9%) − 0.981 (98.1%)	168	94,38	0.910 (91.0%) − 0.978 (97.8%)	
Runny nose – Yes	9	25,00	0.109 (10.9%) − 0.391 (39.1%)	1	6,25	0.05824 (5.82%) − 0.1832 (18.32%)	25	14,04	0.089 (8.9%) − 0.191 (19.1%)	***0,1537 (b)***
Runny nose – No	27	75,00	0.609 (60.9) − 0.891 (89.1%)	15	93,75	0.8428 )84.28%) − 1.0 (100%)	153	85,96	0.809 (80.9%) − 0.911 (91.1%)	
Odynophagia – Yes	8	22,22	0.078 (7.8%) − 0.366 (36.6%)	0	0,00	0 (0.0%)	20	11,24	0.066 (6.6%) − 0.159 (15.9%)	0,0283* (b)
Odynophagia - No	28	77,78	0.645 (64.5%) − 0.911 (91.1%)	16	100,00	1 (100%)	158	88,76	0.841 (84.1%) − 0.934 (93.4%)	
Diarrhea - Yes	0	0,00	0 (0.0%)	4	25,00	0.0805 (8.05%) − 0.4195 (41.95%)	18	10,11	0.057 (5.7%) − 0.145 (14.5%)	0,0053* (b)
Diarrhea - No	36	100,00	1 (100%)	12	75,00	0.5805 (58.05%) − 0.9195 (91.95%)	160	89,89	0.855 (85.5%) − 0.943 (94.3%)	
Asthenia – Yes	4	11,11	0.011 (1.1%) − 0.211 (211%)	1	6,25	0.05824 (5.82%) − 0.1832 (18.32%)	26	14,61	0.094 (9.4%) − 0.198 (19.8%)	***0,5373 (b)***
Asthenia - No	32	88,89	0.812 (81.2%) − 0.966 (96.6%)	15	93,75	0.8428 )84.28%) − 1.0 (100%)	152	85,39	0.802 (80.2%) − 0.906 (90.6%)	
Chest pain – Yes	1	2,78	0.026 (2.6%) − 0.082 (8.2%)	2	12,50	0.0787 (7.87%) − 0.1712 (17.12%)	51	28,65	0.220 (22.0%) − 0.353 (35.3%)	0,0003* (b)
Chest pain - No	35	97,22	0.917(91.7%) − 1.0(100%)	14	87,50	0.769 (76.9%) − 0.981 (98.1%)	127	71,35	0.647 (64.7%) − 0.780 (78.0%)	
Convulsion – Yes	0	0,00	0 (0.0%)	0	0,00	0 (0.0%)	8	4,49	0.015 (1.5%) − 0.075 (7.5%)	***0,1234 (b)***
Convulsion - No	36	100,00	1 (100%)	16	100,00	1 (100%)	170	95,51	0.925 (92.5%) − 0.985 (98.5%)	
Vomiting – Yes	1	2,78	0.026 (2.6%) − 0.082 (8.2%)	0	0,00	0 (0.0%)	3	1,69	0 (0.0%) − 0.0868 (8.68%)	***0,6848 (b)***
Vomiting - No	35	97,22	0.917(91.7%) − 1.0(100%)	16	100,00	1 (100%)	175	98,31	0.940 (94.0%) − 0.993 (99.3%)	
Backache – Yes	0	0,00	0 (0.0%)	0	0,00	0 (0.0%)	3	1,69	0 (0.0%) − 0.0868 (8.68%)	***0,4609 (b)***
Backache - No	36	100,00	1 (100%)	16	100,00	1 (100%)	175	98,31	0.940 (94.0%) − 0.993 (99.3%)	

When evaluating gestational trimester, prenatal care, comorbidities, birth complications, and chest tomography of pregnant women in relation to their outcome, the influence of the disease on specific pregnancy-related comorbidities and the overlap of other pre-existing conditions was detected, as well as greater lung involvement (Table 4).

**Table 4:** Clinical profile of pregnant and postpartum women with a positive test for SARS Cov-2 and the mother’s outcome from April/2020 to June/2022

Gestational Age	Discharge (N = 210)	%	CI 95%	Death (N = 20)	%	CI 95%	*P-valor*
1st Quarter	9	4,29	0.015 (1.5%) − 0.070 (7.0%)	0	0,00	0	***0,4236 (b)***
2nd Quarter	51	24,29	0.185 (18.5%) − 0.301 (30.1%)	3	15,00	0.0904 (9.04%) − 0.2495 (24.95%)	
3rd Quarter	150	71,43	0.653 (65.3%) − 0.775 (77.5%)	17	85,00	0.5336 (53.36%) − 0.9664 (96,64%)	
**Prenatal**	**Discharge (N = 210)**	**%**	**CI 95%**	**Death (N = 20)**	**%**		***P-valor***
Yes	183	87,14	0.826 (82.6%) − 0.917 (91.7%)	17	85,00	0.5336 (53.36%) − 0.9664 (96,64%)	***0,7959 (b)***
No	27	12,86	0.083 (8.3%) − 0.174 (17.4%)	3	15,00	0.0904 (9.04%) − 0.2495 (24.95%)	
**Comorbidities**	**Discharge (N = 210)**	**%**	**CI 95%**	**Death (N = 20)**	**%**		***P-valor***
None	112	53,33	0.466 (46.6%) − 0.601 (60.1%)	5	25,00	0.0215 (2.15%) − 0.2785 (27.85%)	0,0411* (b)
Pregnancy-specific	51	24,29	0.185 (18.5%) − 0.301 (30.1%)	7	35,00	0.2352 (23.52%) − 0.5648 (56.48%)	
Of pregnancy superimposed on other pre-existing	17	8,10	0.044 (4.4%) − 0.118 (11.8%)	1	5,00	0.0012 (0.12%) − 0.0988 (9.88%)	
Pre-existing	30	14,29	0.096 (9.6%) − 0.190 (19.0%)	7	35,00	0.2352 (23.52%) − 0.5648 (56.48%)	
**Birth complications**	**Discharge (N = 210)**	**%**	**CI 95%**	**Death (N = 20)**	**%**		***P-valor***
None	78	54,93	0.306 (30.6%) − 0.437 (43.7%)	7	35,00	0.2352 (23.52%) − 0.5648 (56.48%)	***0,0626 (b)***
Intrauterine Fetal Death	7	4,93	0.009 (0.9%) − 0.058 (5.8%)	0	0,00	0	
Prematurity	57	40,14	0.211 (21.1%) − 0.332 (33.2%)	13	65,00	0.3925 (39.25%) − 0.8575 (85.75%)	
Not applicable	68	32,38	0.261 (26.1%) − 0.387 (38.7%)	0	0	0	
**Range (1st Chest CT - %) (2)**	**Discharge (N = 170)**	**%**	**CI 95%**	**Death (N = 18)**	**%**		***P-valor***
< 20%	33	19,41	0.135 (13.5%) − 0.254 (25.4%)	0	0,00	0	0,0006* (b)
Between 20% and 40%	57	33,53	0.264 (26.4%) − 0.406 (40.6%)	2	11,11	0.0215 (2.15%) − 0.1644 (16.44%)	
>=40%	80	47,06	0.396 (39.6%) − 0.546 (54.6%)	16	88,89	0.4751 (47.51) − 0.9579 (95.79%)	

**Fig. 1:**
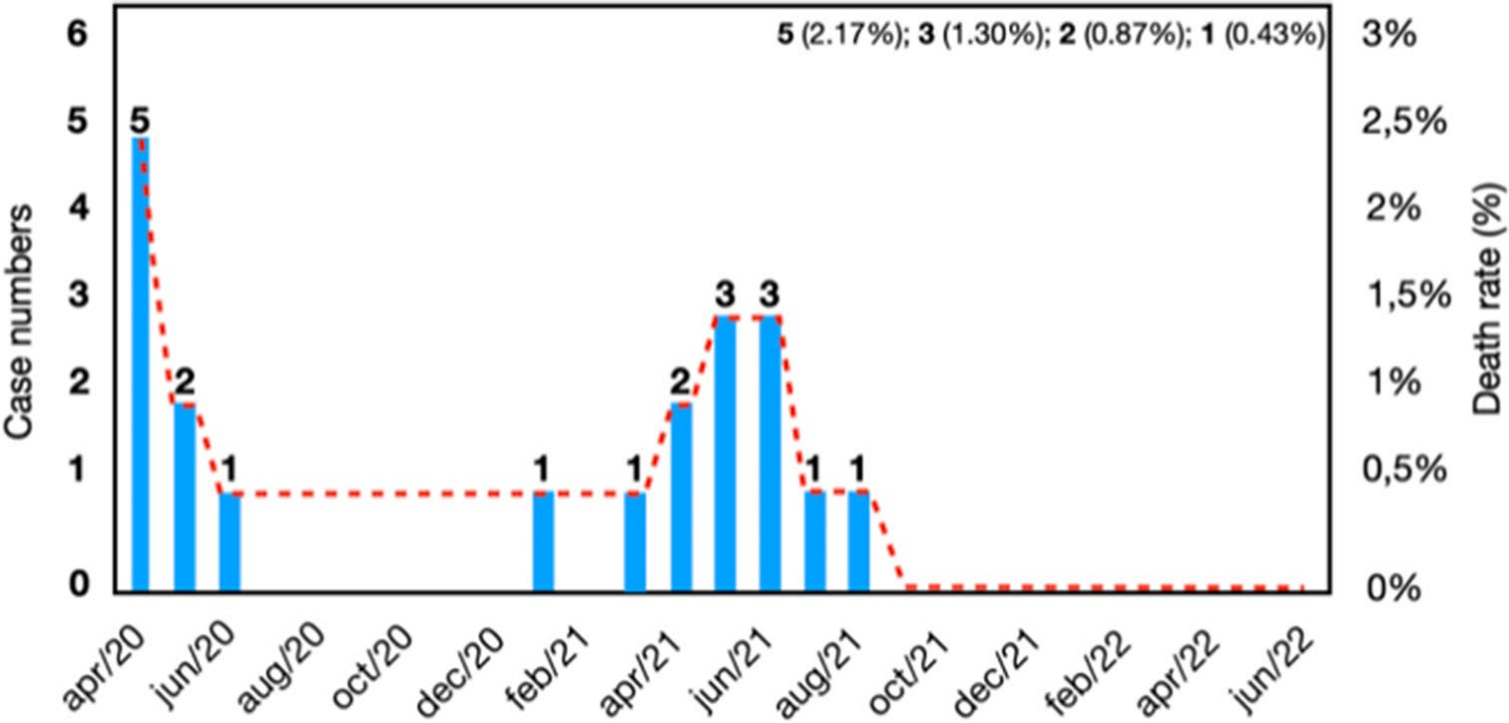
Histogram showing the evolution of the number of case fatality rate of pregnant women with COVID-19 treated at Fundação Santa Casa de Misericórdia do Pará, Belém, from April/2020 to June/2022

Considering the case fatality rate of 8.7% (*n* = 20) from April 2020 to June 2022, an increase in the number of deaths is observed from March 2021 to June 2021, corresponding to the increase in the number of cases, followed by a stabilization from July 2021 onwards. After August 2021 and the end of the study period, no deaths related to SARS-CoV-2 infection were recorded (Fig. 1).

## Discussion

The present study describes the clinical-epidemiological profile and maternal outcome of COVID-19 in pregnant women hospitalized at the main maternal and child hospital in the Brazilian state of Pará, especially during the pre-vaccination period against COVID-19. Regarding age range and ethnicity, most participants were between 20 and 29 years old and self-declared as mixed-race, similar to records made in other Brazilian regions [18, 19]. Furthermore, the highest number of COVID-19 cases attended at FSCMPA was found in women from municipalities in the interior of the State of Pará, considering that the hospital served as a regional reference for high-risk maternal and childcare and for severe cases of COVID-19. It is worth noting that by the end of May 2020, severe cases of COVID-19 were more reported in municipalities in the interior of the states of Pará and Amazonas than in the capitals [20].

In the present study, the highest number of serious cases of COVID-19 were recorded in the late term of pregnancy residents of northern Brazil were similar to records made in other regions of Brazil [21] and in other countries such as the Czech Republic [22], United Kingdom [23] and China [24], suggesting that the attention and care of pregnant women during this period requires maximum attention in relation to COVID-19, as reported by Medeiros et al. [25]. The most frequent comorbidities in this study were systemic arterial hypertension and gestational hypertensive syndromes. High blood pressure and diabetes are clinical conditions that can increase the risk of complications from COVID-19, in addition to obesity, elderly age and chronic lung disease [26].

It is known that pregnancy itself is a physiological event, but the anatomical and physiological changes imposed by this period can exacerbate pre-existing comorbidities and make pregnant women susceptible to various infections, including respiratory ones [26]. SARS-CoV-2 infection during pregnancy can have severe consequences for the fetus, as it relies on maternal oxygenation, and if sufficient oxygen supply does not reach through the placenta, fetal circulation may be compromised. This would explain why pregnant women may progress to preterm labor, have babies with low birth weight, or experience intrauterine growth restriction [27]. However, we observed that the symptoms most related to COVID-19 exacerbation and possible maternal death were dyspnea, headache, anosmia, odynophagia, diarrhea, and chest pain. Interestingly, half of the population in this study required ICU admission, but there is a point to consider [28, 29]. The care profile of the ICU at the maternity hospital of FSCMPA is for women in the perinatal period, which means that patients are admitted daily for management of complications related to this period. Thus, it cannot be ensured that women were admitted to the ICU necessarily due to worsening COVID-19 symptoms, as many of them had comorbidities that, in some situations, would require transfer to the ICU.

The most frequent perinatal complications in this study were preterm birth and fetal death. Prematurity during the most critical period of COVID-19 is mentioned in other studies [30–32]; however, one factor to consider is whether preterm birth was triggered by SARS-CoV-2 infection or by complications in the pregnancy itself. Additionally, it should be noted that due to the high rates of cesarean delivery in the hospital where the study was conducted, it cannot be accurately attributed whether prematurity was due to maternal SARS-CoV-2 infection, like what has been observed in other studies [33–35].

The case fatality rate found in this study was 8.7%, with a higher number of deaths observed between March and June 2021, corresponding to the peak of the pandemic in the Amazon region and Brazil, which was significantly higher than observed in other studies worldwide [7, 8, 11, 36]. However, it is not possible to affirm that case fatality rate was solely due to COVID-19, considering that the majority had specific pregnancy-related comorbidities. The high morbidity and mortality rate from COVID-19 among Brazilian pregnant women may be related to both the pathophysiological conditions inherent in the gestational process and the illness caused by SARS-CoV-2 infection, as well as chronic problems faced by Brazilian obstetric care - such as low-quality prenatal care and difficulty accessing emergency and high-complexity care [35].

From this perspective, Villar et al. [11] observed that deaths were more concentrated in institutions in less developed regions, implying that when ICU services and resources are not fully available, COVID-19 in pregnancy can be lethal. This was corroborated by another study [36], stating that SARS-CoV-2 infection is more frequent in people living in socially and economically disadvantaged environments, which was confirmed by Emeruwa et al. [37]. In this study, a stabilization of cases and deaths was observed from July 2021 onwards, which may be justified by the advent of vaccination in the country. This is because vaccination reduces the risk of developing COVID-19 and the severity of the disease if an advanced infection occurs, as well as reducing the risk of stillbirth. Additionally, all available evidence supports the safety of administering currently available vaccines before, during, and after pregnancy. One limitation of the study is that it was conducted in a single setting, which does not allow for the generalization of the information found here and a comprehensive representation of reality. Furthermore, secondary data sources were used, which may introduce information bias due to incompleteness or underreporting of cases.

## Conclusion

The dynamics of the COVID-19 pandemic in pregnant women in the Brazilian Amazon affect young adult women of non-white ethnicity in the third trimester of pregnancy. Specific pregnancy-related and pre-existing comorbidities associated with SARS-CoV-2 infection directly contribute to the worsening clinical condition, leading to complications such as prematurity, fetal, and maternal death. There was a tendency towards case stabilization after the vaccination period, and the mortality rate was considered low compared to the total sample.

## Data Availability

All related data have been presented within the manuscript. The dataset supporting the conclusions of this article is available from the corresponding author upon request.
